# Estudo descritivo dos eventos supostamente atribuíveis à vacinação contra a mpox no Brasil em 2023

**DOI:** 10.1590/0102-311XPT006624

**Published:** 2024-11-04

**Authors:** Roberta Mendes Abreu Silva, Carla Dinamerica Kobayashi, Adriano Ferreira Martins, Ana Catarina de Melo Araújo, Paulo Henrique Santos Andrade, Martha Elizabeth Brasil da Nóbrega, Cibelle Mendes Cabral, Monica Brauner de Moraes, Felipe Daniel Cardoso, Thayssa Neiva da Fonseca Victer, Amanda Krummenauer, Rodrigo Otávio Pereira Sayago Soares, Eder Gatti Fernandes, Jadher Percio

**Affiliations:** 1 Secretaria de Vigilância em Saúde e Ambiente, Ministério da Saúde, Brasília, Brasil.; 2 Programa de Treinamento em Epidemiologia Aplicada aos Serviços do SUS, Ministério da Saúde, Brasília, Brasil.

**Keywords:** Mpox, Vacinas Atenuadas, Vacinação, Farmacovigilância, Evento Adverso, Mpox, Attenuated Vaccines, Vaccination, Pharmacovigilance, Adverse Event, Mpox, Vacunas Atenuadas, Vacunación, Farmacovigilancia, Evento Adverso

## Abstract

O objetivo deste estudo é descrever as ações de vigilância e segurança da vacina contra a mpox (Jynneos) no Brasil, de 2022 a 2023. Realizou-se estudo descritivo dos casos de mpox e dos eventos supostamente atribuíveis à vacinação e/ou imunização para vacina Jynneos, registrados nos sistemas de informação. Foram calculadas frequências absolutas e relativas e coeficientes de notificação de eventos supostamente atribuíveis à vacinação e/ou imunização por mil doses administradas. Foram registrados 9.596 casos de mpox e distribuídas 49 mil doses de vacina; dessas, 14.395 (31%) doses foram administradas e 65 eventos supostamente atribuíveis à vacinação e/ou imunização (4,5 por mil doses administradas) foram notificados. Todos os eventos supostamente atribuíveis à vacinação e/ou imunização foram não graves e 22 (33,8%) casos apresentaram reações relacionadas ao produto. Os resultados estão dentro da frequência esperada. No entanto, o número de doses administradas pode não ter sido suficiente para detectar eventos raros ou inusitados. Portanto, a vigilância contínua é necessária para garantir a efetividade e a segurança da vacinação, especialmente entre os grupos de maior risco.

## Abstract (en)

This study describes the surveillance and safety actions of the mpox (Jynneos) vaccine in Brazil, from 2022 to 2023. A descriptive study of mpox cases and events supposedly attributable to vaccination and/or immunization for Jynneos vaccine, recorded in the information systems, was conducted. Absolute and relative frequencies and reporting coefficients of events supposedly attributable to vaccination and/or immunization per 1,000 administered doses were calculated. A total of 9,596 cases of mpox were registered and 49,000 doses of vaccine were distributed, of which 14,395 (31%) were administered and 65 events supposedly attributable to vaccination and/or immunization (4.5 per 1,000 administered doses) were notified. All events supposedly attributable to vaccination and/or immunization were non-serious, and 22 (33.8%) cases had product-related reactions. Results are within the expected frequency; however, the number of administered doses may not have been sufficient to detect rare or unusual events. Continued surveillance is necessary to ensure vaccination effectiveness and safety, especially among groups at higher risk for the disease.

## Resumen

El objetivo fue describir las acciones de vigilancia y seguridad de la vacuna contra mpox (Jynneos) en Brasil, del 2022 al 2023. Se realizó un estudio descriptivo de los casos de mpox y de los eventos supuestamente atribuibles a la vacunación o inmunización para la vacuna Jynneos, registrados en los sistemas de información. Se calcularon frecuencias absolutas y relativas y coeficientes de notificación de eventos supuestamente atribuibles a la vacunación o inmunización por 1.000 dosis administradas. Se registraron 9.596 casos de mpox y se distribuyeron 49.000 dosis de vacuna, de estas se administraron 14.395 (31%) dosis y se reportaron 65 eventos supuestamente atribuibles a la vacunación o inmunización (4,5 por 1.000 dosis administradas). Todos los eventos supuestamente atribuibles a la vacunación o inmunización no fueron graves y 22 (33,8%) casos tuvieron reacciones relacionadas con el producto. Los resultados son acordes a la frecuencia esperada. Sin embargo, puede que el número de dosis administradas no haya sido suficiente para detectar eventos raros o inusuales. Por lo tanto, es necesaria una vigilancia continua para garantizar la eficacia y la seguridad de la vacunación, especialmente entre los grupos con mayor riesgo de contraer la enfermedad.

## Introdução

O vírus monkeypox é o agente responsável por uma doença zoonótica cujos sintomas mais comuns são erupções cutâneas ou lesões nas mucosas, acompanhadas de febre, cefaleia, mialgia e linfoadenopatia ^1^ ^,^ ^2^. A doença geralmente evolui para quadros leves e moderados, podendo durar de duas a quatro semanas; no entanto, os pacientes imunodeprimidos podem desenvolver a forma grave da doença, incluindo sepse, desidratação grave, insuficiência respiratória e encefalite ^1^ ^,^ ^2^ ^,^ ^3^ ^,^ ^4^.

O diagnóstico é feito por meio da busca de partículas virais nas lesões cutâneas e não há um tratamento antiviral específico para a doença, sendo os quadros sintomáticos tratados com cuidados de suporte e com o uso do medicamento tecovirimat ^1^ ^,^ ^2^. A prevenção contra a doença inclui o isolamento do paciente durante o período de transmissibilidade, a busca ativa dos seus contatos para o diagnóstico oportuno, a interrupção da cadeia de transmissão e, mais recentemente, a vacinação contra o mpox ^1^ ^,^ ^2^.

No Brasil, diversos esforços foram realizados para identificar medidas de prevenção e controle da doença ^5^. Entre eles, a vacina contra a mpox ^6^, implementada no Brasil e utilizada em um cenário de mundo real pela primeira vez durante o surto de mpox em 2022 ^2^. Trata-se de uma vacina de vírus vivo, produzida a partir de um Orthopoxvirus atenuado e não replicante. Essa vacina é, até o momento, a única aprovada pelas autoridades regulatórias para a prevenção da mpox ^7^ ^,^ ^8^ ^,^ ^9^. 

No geral, a vacina apresenta bom perfil de segurança, com esquema vacinal de duas doses, e é licenciada para a utilização em adultos, sendo administrada com intervalo de 28 dias por via subcutânea, fornecendo proteção com até duas semanas após a segunda dose ^7^ ^,^ ^8^ ^,^ ^9^ ^,^ ^10^ ^,^ ^11^.

A vacinação contra a mpox (Jynneos) começou em 13 de março de 2023, no Brasil, e seu objetivo principal era proteger os indivíduos com maior risco ou vulnerabilidade para a doença, abrangendo estratégias de pré-exposição para pessoas vivendo com HIV/aids e profissionais com maior risco de exposição ocupacional e pós-exposição em pessoas que tiveram contato de risco com casos suspeitos ou confirmados de mpox ^2^ ^,^ ^12^.

Diante desse contexto, torna-se importante descrever as ações de vigilância e a ocorrência de eventos supostamente atribuíveis à vacinação e/ou imunização para vacina contra a mpox (Jynneos), durante o período de implementação e utilização no Brasil. Ao fornecer informações detalhadas sobre a vacinação em um cenário de mundo real, especialmente entre os grupos de maior risco ou vulnerabilidade para a doença, este trabalho contribui para aprimorar estratégias de prevenção e controle da mpox, bem como fornece informações sobre a segurança da vacinação em sua fase pós-autorização.

Logo, este trabalho tem o objetivo de descrever eventos supostamente atribuíveis à vacinação contra a mpox no Brasil em 2023.

## Métodos

### Delineamento

Foi realizado um estudo descritivo de uma série de casos, incluindo casos de mpox e de eventos supostamente atribuíveis à vacinação e/ou imunização, no âmbito das ações de vigilância e segurança da vacinação contra a mpox (Jynneos) no Brasil, 2023.

### Contexto

O Informe técnico operacional de vacinação contra o mpox, publicado em 6 de março de 2023, orienta a estratégia de vacinação nacional executada pelos municípios em conjunto com os estados ^13^.

O registro de doses administradas é realizado no sistema de informação do Programa Nacional de Imunizações (PNI), possibilitando a identificação e o acompanhamento da pessoa vacinada, além do rastreamento da vacina, do lote, do fabricante e da dose administrada.

Com relação à segurança da vacinação, há, no país, o Sistema Nacional de Vigilância de Eventos Supostamente Atribuíveis à Vacinação e/ou Imunização, que reúne quaisquer ocorrências médicas indesejadas após a vacinação, com relação temporal ao uso de uma vacina ou outro imunobiológico (imunoglobulinas e soros heterólogos), não possuindo, necessariamente, uma relação causal com o produto.

Esse sistema recebe notificações de eventos supostamente atribuíveis à vacinação e/ou imunização dos profissionais de saúde nas unidades notificadoras em todo o território nacional. Todos os eventos supostamente atribuíveis à vacinação e/ou imunização graves, raros e inusitados, além dos erros de imunização (programáticos), são de notificação compulsória e pode ser realizada por qualquer profissional de saúde no sistema de informação e-SUS Notifica (https://notifica.saude.gov.br/onboard) - módulo *online* de eventos supostamente atribuíveis à vacinação e/ou imunização -, permitindo o monitoramento da segurança das vacinas ^14^, incluindo os eventos relacionados à vacina contra a mpox (Jynneos).

### Participantes

Foram elegíveis para este estudo todos os indivíduos que tiveram ao menos uma dose da vacina contra a mpox (Jynneos) administrada entre 15 de março de 2023 e 8 de julho de 2023. Adicionalmente, incluiu-se o número de casos notificados e confirmados para a mpox que foram publicados pelo Ministério da Saúde no mesmo período.

Considerou-se caso confirmado: indivíduo com resultado “positivo ou detectável” para o vírus monkeypox por diagnóstico molecular.

### Variáveis

Variáveis sociodemográficas dos casos de eventos supostamente atribuíveis à vacinação e/ou imunização notificados: sexo; faixa etária em anos (18-29; 30-39; 40-49; 50-59 e ≥ 60); Unidade da Federação (UF); raça/cor da pele (amarela, branca, parda, preta, ignorado).

Variáveis vacinais e clínicas: doses administradas; via de administração; vacina coadministrada (não, sim); tipo de evento (erro de imunização e evento adverso); gravidade (eventos supostamente atribuíveis à vacinação e/ou imunização não grave, grave e óbito); causalidade (A: associações consistentes com a vacina; B: associações indeterminadas; C: associação inconsistente ou coincidente; D: inclassificável). Todos os eventos supostamente atribuíveis à vacinação e/ou imunização graves foram revisados federalmente para reavaliação da classificação de causalidade. Foi considerado evento supostamente atribuível à vacinação e/ou imunização grave qualquer evento clinicamente relevante que: (1) requeira hospitalização; (2) possa comprometer o paciente, ou seja, que ocasione risco de morte e que exija intervenção clínica imediata para evitar o óbito; (3) cause disfunção significativa e/ou incapacidade permanente; (4) resulte em anomalia congênita; e (5) ocasione o óbito ^14^.

### Fonte de dados e mensuração

Para contextualização do cenário epidemiológico, vacinal e de notificações de eventos supostamente atribuíveis à vacinação e/ou imunização, foram utilizadas três fontes de dados, com suas respectivas temporalidades: (1) banco de notificações dos casos de mpox obtidos nos sistemas de notificações (e-SUS Sistema de Informação de Agravos de Notificação, CeVesp e e-SUS Vigilância em Saúde) e do banco de dado legado na plataforma REDCap (https://redcapbrasil.com.br), até 8 de julho de 2023 - Semana Epidemiológica 27; (2) banco de doses administradas por meio da Rede Nacional de Dados em Saúde atualizado em 8 de julho de 2023; (3) banco de notificações de eventos supostamente atribuíveis à vacinação e/ou imunização extraídas por meio do sistema de informação *online* e-SUS Notifica, atualizado em 8 de julho de 2023.

O cálculo da incidência foi baseado no número de casos confirmados da doença como numerador e a população estimada pelo Instituto Brasileiro de Geografia e Estatística (IBGE) ^15^ em 2021 como denominador, expressa por 100 mil habitantes.

O coeficiente de notificação de eventos supostamente atribuíveis à vacinação e/ou imunização pela vacina foi calculado considerando como numerador os eventos supostamente atribuíveis à vacinação e/ou imunização notificados para a vacina e, no denominador, o total de doses administradas por mil. Foram consideradas notificações de interesse aquelas que apresentaram o código imunobiológico da vacina e excluídas da análise as notificações com situação cancelada.

### Controle de viés

Para controlar o viés na avaliação dos eventos supostamente atribuíveis à vacinação e/ou imunização, optou-se por estratificar a população do estudo segundo doses administradas. Essa abordagem permitiu uma análise mais precisa dos dados, considerando potenciais variações nos eventos notificados em relação à dose da vacina e à avaliação de causalidade, mitigando o risco de viés de classificação.

### Tamanho do estudo

Trabalhou-se com o total de indivíduos que tiveram notificação de mpox no Brasil, bem como aqueles que apresentaram ao menos uma dose administrada da vacina (Jynneos) e que tiveram notificação de eventos supostamente atribuíveis à vacinação e/ou imunização no período avaliado.

### Métodos estatísticos

A análise dos dados foi realizada por meio de medidas de frequência relativa, absoluta e coeficiente de notificação. Para estimar a incidência da doença, foi considerado o número de casos de mpox no numerador e a população estimada em 2021 no denominador, por 100 mil habitantes. Os coeficientes de notificação dos eventos supostamente atribuíveis à vacinação e/ou imunização foram calculados utilizando como numerador o número de notificações de eventos supostamente atribuíveis à vacinação e/ou imunização e como denominador as doses administradas para a variável correspondente. Para o processamento dos dados, foi utilizado o Microsoft Excel 2016 (https://products.office.com/).

### Aspectos éticos

Por serem secundários para fins de vigilância epidemiológica em saúde, sem possibilidade de identificação individual, o estudo está consoante a Resolução nº 674, de 6 de maio de 2022, do Conselho Nacional de Saúde dispensando-se a apreciação pelo sistema do Comitê de Ética em Pesquisa.

## Resultados

**Figura 1:**
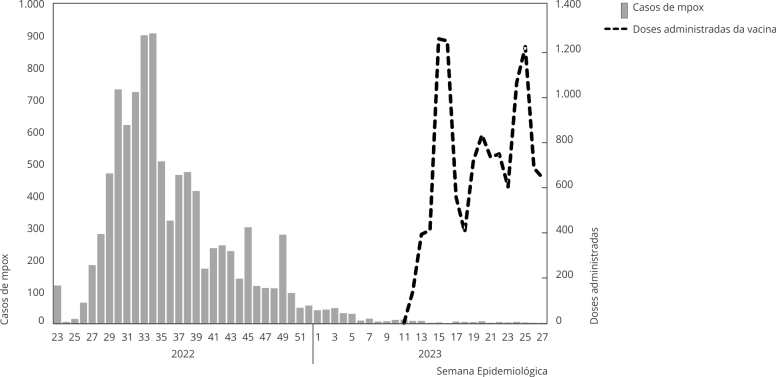
Distribuição da frequência de casos confirmados segundo Semana Epidemiológica de notificação de mpox e de doses administradas da vacina. Brasil, 2022-2023.

Desde o primeiro caso confirmado de mpox no Brasil, em 9 de junho de 2022, até o início da vacinação, em 13 de março de 2023, foram confirmados 9.596 casos da doença viral no país, incluindo 15 óbitos. Após o início da vacinação, até 8 de julho de 2023, foram confirmados 92 casos de mpox, incluindo um óbito; totalizando 9.688 casos e 16 óbitos confirmados nesse período (Figura 1).

O maior número de casos de mpox foi observado entre as Semanas Epidemiológicas 33 a 35 de 2022, enquanto a vacinação começou na Semana Epidemiológica 11 de 2023, com o maior número de doses administradas na Semana Epidemiológica 14 de 2023 (Figura 1).

**Figura 2:**
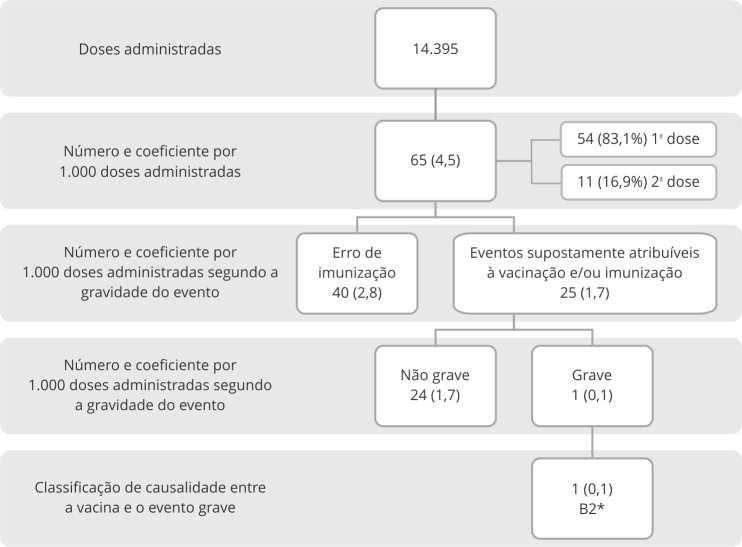
Descrição das doses administradas e incidência de eventos supostamente atribuíveis à vacinação e/ou imunização para a vacina contra a mpox por tipo de evento, gravidade e causalidade. Brasil, março a julho de 2023.

No que diz respeito ao monitoramento realizado pela farmacovigilância pós-autorização emergencial, ao todo foram identificadas 65 notificações de eventos supostamente atribuíveis à vacinação e/ou imunização. Foram administradas 14.395 doses dessa vacina (D1: 9.794; D2: 4.601; taxa de abandono 53%), sendo a incidência total de eventos supostamente atribuíveis à vacinação e/ou imunização de 4,5 notificações por mil doses administradas (Figura 2). Do total de casos notificados, 40 (61,5%) foram erros de imunização e 25 casos de eventos supostamente atribuíveis à vacinação e/ou imunização. Apenas um foi considerado grave, sem ocorrência de óbito.

* Dados da investigação são conflitantes em relação à causalidade.

Em relação às características sociodemográficas, observa-se que a maior parte dos casos foi notificada pelos estados de São Paulo e do Rio de Janeiro. Cerca de 75,9% das pessoas notificadas eram do sexo masculino, 29,6% na faixa etária 30 a 39 anos (Tabela 1).

**Tabela 1:** Distribuição das notificações de eventos supostamente atribuíveis à vacinação e/ou imunização para a vacina contra a mpox por variáveis sociodemográficas. Brasil, março a julho de 2023.

Variáveis	n	%	Doses administradas	Coeficiente (por mil doses administradas)
Brasil	65	100,0	14.935	4,5
Unidade da Federação				
Bahia	11	16,9	233	47,2
Distrito Federal	1	1,5	332	3,0
Minas Gerais	1	1,5	1.567	0,6
Pará	3	4,6	247	12,1
Rio de Janeiro	22	33,8	2.306	9,5
Rio Grande do Sul	3	4,6	563	5,3
São Paulo	24	36,9	4.605	5,2
Sexo				
Feminino	21	24,1	1.447	14,5
Masculino	44	75,9	12.948	3,4
Faixa etária (anos)				
18-29	12	16,7	2.573	4,7
30-39	21	29,6	4.259	4,9
40-49	17	25,9	3.934	4,3
50-59	9	14,8	2.500	3,6
≥ 60	6	13	1.126	5,3
Raça/Cor da pele *				
Amarela	9	16,7	-	-
Branca	24	51,9	-	-
Parda	6	11,1	-	-
Preta	6	9,3	-	-
Ignorado	17	11,1	-	-

* Não há registro de doses administradas segundo variável raça/cor da pele.

Os maiores coeficientes de notificações foram para o Estado da Bahia (47,2 eventos supostamente atribuíveis à vacinação e/ou imunização por mil doses administradas), sexo feminino (14,5 eventos supostamente atribuíveis à vacinação e/ou imunização por mil doses administradas) e na faixa etária maior ou igual a 60 anos (5,3 eventos supostamente atribuíveis à vacinação e/ou imunização por mil doses administradas) (Tabela 1).

Entre as notificações, 33,8% foram classificadas como A1, isto é, reações relacionadas ao produto, causada ou precipitada pela vacina, ou por um ou mais dos componentes da vacina. Observa-se, também, que 98,5% das notificações não apresentavam outras vacinas coadministradas e, em 72,3% dos casos, a via de administração foi subcutânea e a utilização de vacina vencida foi o principal termo relatado como erro de imunização, o que se refere aos frascos não utilizados até quatro semanas após o descongelamento (Tabela 2).

**Tabela 2:** Descrição da causalidade, coadministração de vacinas e vias de administração das notificações de eventos supostamente atribuíveis à vacinação e/ou imunização por dose da vacina contra mpox. Brasil, março a julho de 2023.

	1ª dose (n = 54)		2ª dose (n = 11)		Total	
	n	%	n	%	n	%
Causalidade						
A1 - Reação relacionada ao produto	21	38,9	1	9,1	22	33,8
A3 - Reação relacionada a erro de imunização	1	1,9	0	-	1	1,5
B2 - Dados conflitantes em relação à causalidade	1	1,9	0	-	1	1,5
Erros de imunização sem ocorrência de eventos supostamente atribuíveis à vacinação e/ou imunização	31	57,4	10	90,9	41	63,1
Utilização de vacina vencida *	24	44,4	0	-	24	36,9
Via incorreta de vacinação	5	9,3	9	81,8	14	21,5
Contraindicação à vacinação	2	3,7	0	-	2	3,1
Vacina coadministrada						
Não	54	100,0	10	90,9	64	98,5
Sim	0	-	1	9,1	1	1,5
Via de administração						
Intramuscular	8	14,8	9	81,8	17	26,2
Subcutânea	46	85,2	1	9,1	47	72,3
Ignorado	0	-	1	9,1	1	1,5

* Refere-se aos frascos não utilizados até quatro semanas após o descongelamento.

Para as notificações de eventos supostamente atribuíveis à vacinação e/ou imunização, os principais sinais e sintomas descritos foram dor, edema, cefaleia, febre e mialgia (Tabela 3). A maioria dos casos (96%) iniciou sinais e sintomas em menos de 24 horas após a vacinação.

**Tabela 3:** Descrição dos termos nas notificações de eventos supostamente atribuíveis à vacinação e/ou imunização para a vacina contra a mpox. Brasil, março a julho de 2023.

	1ª dose (n = 54)		2ª dose (n = 11)		Total	
	n	%	n	%	n	%
Sinais e sintomas sistêmicos						
Dor	7	13,0	0	-	7	10,8
Edema	7	13,0	0	-	7	10,8
Febre	3	5,6	1	9,1	4	6,2
Mialgia	3	5,6	0	-	3	4,6
Diarreia	3	5,6	0	-	3	4,6
Convulsão	2	3,7	0	-	2	3,1
Fadiga	2	3,7	0	-	2	3,1
Dor lombar	1	1,9	0	-	1	1,5
Eritema	1	1,9	0	-	1	1,5
Hiperemia	1	1,9	0	-	1	1,5
Linfonodos axilares aumentados	1	1,9	0	-	1	1,5
Manchas na pele	1	1,9	0	-	1	1,5
Petéquias	1	1,9	0	-	1	1,5
Prurido	1	1,9	0	-	1	1,5
Sinais e sintomas no local da injeção						
Dor localizada	3	5,6	0	-	3	4,6
Dor no local da injeção	2	3,7	0	-	2	3,1
Hematoma local da injeção	1	1,9	0	-	1	1,5
Endurecimento cutâneo	1	1,9	0	-	1	1,5
Nódulo cutâneo	1	1,9	0	-	1	1,5
Nódulo doloroso	1	1,9	0	-	1	1,5
Rubor	1	1,9	0	-	1	1,5
Edema dos braços	0	-	1	9,1	1	1,5

O caso de eventos supostamente atribuíveis à vacinação e/ou imunização grave teve sua causalidade classificada como B2, isto é, os dados da investigação são conflitantes em relação à causalidade. Tratava-se de um paciente vivendo com HIV que apresentou crise convulsiva 48 horas após a administração da vacina, com observação hospitalar por até 24 horas. Apesar da relação temporal com a vacinação, outros diagnósticos diferenciais, comuns em pacientes imunocomprometidos não foram investigados e/ou descartados. Em avaliação pela esfera federal, foi mantida a causalidade classificada pelo estado; no entanto, por não atender aos critérios de gravidade descritos no manual de eventos supostamente atribuíveis à vacinação e/ou imunização, o caso foi reclassificado como não grave.

## Discussão

Este é o primeiro estudo a documentar e descrever os resultados da vacinação contra a mpox no Brasil, com foco na segurança da vacina, após o monitoramento realizado pela farmacovigilância nos serviços do Sistema Único de Saúde (SUS). Este estudo aponta que a distribuição dos casos confirmados de mpox e o início da vacinação no Brasil ocorreram em momentos distintos no período avaliado. Adicionalmente, os dados de farmacovigilância indicam que o número de notificações de eventos supostamente atribuíveis à vacinação e/ou imunização para a vacina contra a mpox (Jynneos) foi menor do que o esperado (> 10% de reações classificadas como comuns). Não foi registrada ocorrência de eventos supostamente atribuíveis à vacinação e/ou imunização grave ou de óbito após a vacinação, e, a partir dos sinais e sintomas avaliados, não foi identificado nenhum sinal de segurança novo ou inusitado para essa vacina.

De forma geral, os resultados indicam que a vacinação começou após 15 meses do início da epidemia de mpox no país, quando a curva epidêmica já tinha alcançado seu pico e passado pelo período de declínio, apresentando poucos casos notificados semanalmente a partir de então.

Esse lapso temporal pode ser justificado por dois principais motivos: (1) os trâmites operacionais de importação e envio dos produtos pelo fabricante, incluindo a análise de qualidade realizada pelo Instituto Nacional de Controle de Qualidade em Saúde, Fundação Oswaldo Cruz (INCQS/Fiocruz) antes da liberação para uso; e (2) o tempo que foi necessário para a elaboração e a aprovação ética dos protocolos de pesquisa, já que o plano original era usar as vacinas disponíveis para realizar estudos de efetividade e segurança. O uso das vacinas para a prevenção em grupos prioritários só foi aventado como uma como ação de saúde pública após o resultado da Comissão Nacional de Ética em Pesquisa, em janeiro de 2023. Com isso, o PNI planejou e implantou a vacinação, com os estados e municípios, em aproximadamente dois meses, se considerarmos o tempo entre a mudança da estratégia e o início da vacinação propriamente dita.

No entanto, apesar de o número de casos confirmados ter reduzido expressivamente em 2023, a identificação de novos casos em todas as semanas epidemiológicas subsequentes apontam que o vírus mpox permanece circulante no Brasil. Logo, a manutenção da vacinação para a população alvo e a garantia de estoque estratégico no país permitem a continuidade de estratégias para a prevenção da doença e controle de surtos ^13^, o que se torna ainda mais relevante ao se considerar um contexto de baixa adesão à vacinação.

A pouca adesão à vacinação pode ser evidenciada pela baixa frequência de doses administradas em relação ao total de doses adquiridas (35,9%). Esse fenômeno pode ser explicado, em partes, pela complacência que ocorre quando os riscos percebidos de infecção e adoecimento são baixos e a vacinação não é considerada importante pela população-alvo ^16^. A hesitação vacinal (recusa ou atraso na aceitação de vacinas) é influenciada pela complacência, mas também por outros fatores, incluindo a conveniência e a confiança na efetividade e segurança das vacinas ^16^.

O monitoramento da segurança da vacina no Brasil, até o momento, não identificou eventos novos ou inesperados, que poderiam indicar sinais de segurança para essa vacina. O coeficiente de notificação está dentro do esperado e o perfil de segurança da vacina encontrado foi consistente com os estudos pré-licenciamento ^6^ ^,^ ^7^ ^,^ ^8^ ^,^ ^9^. Os eventos adversos de saúde mais comuns relatados foram não graves e incluíram reações no local da injeção.

Ao avaliar o cenário de distribuição das notificações de eventos supostamente atribuíveis à vacinação e/ou imunização no território nacional, os maiores quantitativos de notificações foram identificados nos estados de São Paulo e do Rio de Janeiro. É importante destacar que maiores quantitativos não implicam em causalidade. Esses indicadores permitem mensurar a sensibilidade de notificações das vigilâncias estaduais, relacionada às notificações de eventos não graves e graves, e são diretamente influenciados pelo denominador de doses administradas. Logo, as interpretações devem ser avaliadas com cautela.

A maior frequência de notificação para indivíduos do sexo masculino e na faixa etária entre 30 a 49 anos pode ser justificada pelo perfil sociodemográfico dos grupos prioritários para a vacinação, que incluem pessoas vivendo com HIV/aids. Sabe-se que, em 2021, a razão de sexo da taxa de detecção de aids por 100 mil habitantes foi de 2,5 entre pessoas do sexo masculino para cada pessoa do sexo feminino, e o maior número de casos de pessoas vivendo com HIV/aids foi entre os adultos jovens ^17^ ^,^ ^18^.

Entre os casos de mpox no mundo ^19^, a transmissão do vírus ocorreu por meio de contato sexual, afetando principalmente homens que fazem sexo com homens. Estudos apontam que pessoas vivendo com HIV/aids foram afetadas de forma desproporcional, representando aproximadamente 42% dos casos de mpox ^20^ ^,^ ^21^. Contudo, ainda não há estudos conclusivos sobre como a infecção pelo HIV afeta o risco de uma pessoa adquirir mpox. Logo, se faz necessário pesquisas adicionais que determinem a contribuição relativa ao comportamento sexual, acesso aos cuidados de saúde sexual e ao risco biológico ^4^.

Observa-se que, na literatura científica, ainda existem lacunas sobre o risco de eventos supostamente atribuíveis à vacinação e/ou imunização em indivíduos com HIV/aids, especialmente considerando que esse grupo é o público-alvo preferencial dessa vacina. Para aprofundar a avaliação dos eventos supostamente atribuíveis à vacinação e/ou imunização nessas pessoas, seria crucial ter acesso a dados clínicos e laboratoriais abrangentes, como tempo desde o diagnóstico, uso de terapia antirretroviral (TARV), contagem de linfócitos CD4+, carga viral e presença de comorbidades. Mesmo diante da falta desses dados para análise, é importante reconhecer e comentar sobre esse aspecto, destacando a necessidade de mais pesquisas e considerações específicas para esse subgrupo de pacientes. Contudo, os resultados da farmacovigilância mostram que os benefícios das vacinas superam os riscos da vacinação dessa população.

Foi identificado que a maior parte das notificações eram referentes a erros isolados de imunização, isto é, sem o risco de eventos supostamente atribuíveis à vacinação e/ou imunização. Entre eles, destacam-se a via de administração incorreta e a administração de vacinas vencidas. O monitoramento de segurança da vacina realizado pelo Centro de Controle e Prevenção de Doenças dos Estados Unidos (CDC) aponta que os erros relacionados à via de administração intradérmica foram relatados com maior frequência nos Estados Unidos, quando a recomendação é administração por via subcutânea ^22^. Considerações clínicas provisórias indicam que a ausência de pápulas sem vazamento da vacina pode ser considerada como administração válida ^23^.

Após a identificação do erro de imunização relativo ao termo “vacina vencida”, foram avaliadas todas as notificações e as descrições do evento para compreender essa ocorrência. Observou-se que o termo era aplicado considerando as orientações do informe técnico operacional de vacinação contra a mpox, que indica que, uma vez descongelada, a vacina tinha validade de até quatro semanas se conservada a +2ºC a +8ºC ^13^. Vale ressaltar que, apesar da identificação dos erros de imunização, eles apresentam uma baixa ocorrência (2,8 a cada mil doses administradas). Os erros de imunização continuam a ser monitorados a fim de garantir práticas seguras na vacinação.

A maior parte das notificações de eventos supostamente atribuíveis à vacinação e/ou imunização foram não graves, com sinais e sintomas de dor, edema, cefaleia, febre e mialgia como os principais termos observados. De forma geral, todos os sinais e sintomas compõem o escopo de eventos esperados para a vacina na literatura ^8^ ^,^ ^24^ ^,^ ^25^ ^,^ ^26^ ^,^ ^27^ ^,^ ^28^ ^,^ ^29^. Em contrapartida, os eventos adversos graves têm baixa frequência e não estão, em sua maioria, relacionados à vacina. Até o momento, nenhum óbito esteve associado diretamente à vacina na literatura ^25^ ^,^ ^26^ ^,^ ^27^ ^,^ ^28^ ^,^ ^30^.

Sabe-se que nem todos os eventos supostamente atribuíveis à vacinação e/ou imunização que ocorreram após a vacinação são causados pela vacina. Nessa avaliação, 33,8% das notificações foram classificadas como A1, isto é, reações relacionadas ao produto, causada ou precipitada pela vacina ou por um ou mais dos seus componentes. A literatura aponta que não foram identificados eventos supostamente atribuíveis à vacinação e/ou imunização graves com relação causal com a vacinação. No entanto, considerando que as vacinas contra a varíola, de gerações anteriores, estiveram associadas ao risco aumentado de miocardite, pericardite e encefalites, é necessário monitorar esses eventos adversos de interesse especial para a vacina contra a mpox (Jynneos) ^31^.

Este artigo está sujeito a limitações devido aos sistemas nacionais de informação de casos de mpox e ao sistema nacional de vigilância de eventos supostamente atribuíveis à vacinação e/ou imunização, que são passivos e podem apresentar subnotificações. eventos supostamente atribuíveis à vacinação e/ou imunização não graves, como reações no local da injeção, não são de notificação compulsória e têm menor probabilidade de serem relatados em comparação com eventos supostamente atribuíveis à vacinação e/ou imunização graves. Outra limitação diz respeito ao baixo número de doses administradas que impacta na identificação de eventos supostamente atribuíveis à vacinação e/ou imunização raros, muito raros ou de interesse especial, incluindo: distúrbios cardíacos (miocardite, pericardite), distúrbios tromboembólicos e neurovasculares ^6^ ^,^ ^8^ ^,^ ^11^.

No que diz respeito às diferenças nas taxas de incidência de eventos supostamente atribuíveis à vacinação e/ou imunização entre as doses da vacina, é cientificamente plausível que essas taxas possam variar ao longo do esquema de vacinação, devido a diversos fatores, como a natureza da resposta imunológica induzida por cada dose ou características específicas dos eventos adversos associados a cada uma delas. Essa variação nas taxas de incidência pode distorcer a percepção do risco real de eventos supostamente atribuíveis à vacinação e/ou imunização, tornando necessário um cuidadoso ajuste na interpretação dos dados.

Além disso, é importante considerar a associação plausível entre a hesitação vacinal - indiretamente aferida pela taxa de abandono ou o não comparecimento para a segunda dose - e os relatos de eventos supostamente atribuíveis à vacinação e/ou imunização. Indivíduos que estão mais hesitantes em receber a vacina podem ser mais propensos a relatar eventos adversos, mesmo que sejam leves ou coincidentes. Da mesma forma, aqueles que não retornam para a segunda dose podem estar mais inclinados a relatar eventos supostamente atribuíveis à vacinação e/ou imunização, especialmente se tiverem experimentado algum evento adverso após a primeira dose. Essa associação entre hesitação vacinal/não comparecimento para a segunda dose e relato de eventos adversos pode distorcer a percepção do risco global de eventos supostamente atribuíveis à vacinação e/ou imunização, podendo levar à sua superestimação.

Ademais, as diferenças nas taxas de notificação de eventos supostamente atribuíveis à vacinação e/ou imunização entre os estados podem refletir as diversas capacidades e níveis de amadurecimento dos sistemas de vigilância e de imunização em cada localidade. Essas disparidades destacam a importância de fortalecer os sistemas de vigilância e imunização em todo o país, para garantir detecção e notificação precisas e oportunas de eventos supostamente atribuíveis à vacinação e/ou imunização.

Portanto, ao analisar e interpretar estudos sobre a segurança da vacinação contra a mpox, é essencial reconhecer e explicitar essas limitações. A compreensão desses fatores pode contribuir para uma interpretação mais precisa dos dados e das conclusões, auxiliando na tomada de decisões e na comunicação efetiva sobre a segurança das vacinas.

De forma geral, os resultados preliminares da vigilância da segurança da vacina pós-autorização emergencial são consistentes com aqueles observados nos ensaios clínicos e apoiam a segurança dessa vacina. O sistema nacional de vigilância de eventos supostamente atribuíveis à vacinação e/ou imunização continuará o monitoramento, e as unidades notificadoras devem continuar a notificar os eventos supostamente atribuíveis à vacinação e/ou imunização graves no e-SUS Notifica para subsidiar novas discussões.
